# Antibiotic prescribing for upper respiratory tract infections and acute bronchitis: a longitudinal analysis of general practitioner trainees

**DOI:** 10.1093/fampra/cmac052

**Published:** 2022-05-28

**Authors:** Emma J Baillie, Gregory Merlo, Parker Magin, Amanda Tapley, Katie J Mulquiney, Joshua S Davis, Alison Fielding, Andrew Davey, Elizabeth Holliday, Jean Ball, Neil Spike, Kristen FitzGerald, Mieke L van Driel

**Affiliations:** General Practice Clinical Unit, The University of Queensland, Brisbane, QLD 4006, Australia; General Practice Clinical Unit, The University of Queensland, Brisbane, QLD 4006, Australia; School of Medicine and Public Health, University of Newcastle, Callaghan, NSW, Australia; GP Synergy NSW & ACT Research and Evaluation Unit, Newcastle, NSW, Australia; School of Medicine and Public Health, University of Newcastle, Callaghan, NSW, Australia; GP Synergy NSW & ACT Research and Evaluation Unit, Newcastle, NSW, Australia; School of Medicine and Public Health, University of Newcastle, Callaghan, NSW, Australia; GP Synergy NSW & ACT Research and Evaluation Unit, Newcastle, NSW, Australia; School of Medicine and Public Health, University of Newcastle, Callaghan, NSW, Australia; School of Medicine and Public Health, University of Newcastle, Callaghan, NSW, Australia; GP Synergy NSW & ACT Research and Evaluation Unit, Newcastle, NSW, Australia; School of Medicine and Public Health, University of Newcastle, Callaghan, NSW, Australia; GP Synergy NSW & ACT Research and Evaluation Unit, Newcastle, NSW, Australia; School of Medicine and Public Health, University of Newcastle, Callaghan, NSW, Australia; Hunter Medical Research Institute, Clinical Research Design and Statistical Support Unit (CReDITSS), New Lambton Heights, NSW 2305, Australia; Eastern Victoria General Practice Training, Regional Training Organisation, Hawthorn, VIC 3122, Australia; University of Melbourne, Department of General Practice and Primary Health Care, Berkeley Street, Carlton, VIC 3053, Australia; Monash University, School of Rural Health, Wellington Road, Clayton, VIC 3800, Australia; University of Tasmania, Tasmanian School of Medicine, Hobart, TAS 7000, Australia; General Practice Training Tasmania, Regional Training Organisation, Hobart, TAS 7000, Australia; General Practice Clinical Unit, The University of Queensland, Brisbane, QLD 4006, Australia

**Keywords:** antibacterial agents, antimicrobial stewardship, general practitioners, graduate medical education, inappropriate prescribing, respiratory tract infections

## Abstract

**Background:**

Most antibiotic prescribing for upper respiratory tract infections (URTIs) and acute bronchitis is inappropriate. Substantive and sustained reductions in prescribing are needed to reduce antibiotic resistance. Prescribing habits develop early in clinicians’ careers. Hence, general practice (GP) trainees are an important group to target.

**Objectives:**

We aimed to establish temporal trends in antibiotic prescribing for URTIs and acute bronchitis/bronchiolitis by Australian GP trainees (registrars).

**Methods:**

A longitudinal analysis, 2010–2019, of the Registrars Clinical Encounters in Training (ReCEnT) dataset. In ReCEnT, registrars record clinical and educational content of 60 consecutive consultations, on 3 occasions, 6 monthly. Analyses were of new diagnoses of URTI and acute bronchitis/bronchiolitis, with the outcome variable a systemic antibiotic being prescribed. The independent variable of interest was year of prescribing (modelled as a continuous variable).

**Results:**

28,372 diagnoses of URTI and 5,289 diagnoses of acute bronchitis/bronchiolitis were recorded by 2,839 registrars. Antibiotic prescribing for URTI decreased from 24% in 2010 to 12% in 2019. Prescribing for acute bronchitis/bronchiolitis decreased from 84% to 72%. “Year” was significantly, negatively associated with antibiotic prescribing for both URTI (odds ratio [OR] 0.90; 95% confidence interval [CI]: 0.88–0.93) and acute bronchitis/bronchiolitis (OR 0.92; 95% CI: 0.88–0.96) on multivariable analysis, with estimates representing the mean annual change.

**Conclusions:**

GP registrars’ prescribing for URTI and acute bronchitis/bronchiolitis declined over the 10-year period. Prescribing for acute bronchitis/bronchiolitis, however, remains higher than recommended benchmarks. Continued education and programme-level antibiotic stewardship interventions are required to further reduce registrars’ antibiotic prescribing for acute bronchitis/bronchiolitis to appropriate levels.

**Key messages**

- GP registrars’ antibiotic prescribing for URTI and acute bronchitis has reduced.
- Registrar URTI/acute bronchitis antibiotics prescribing is less than other GPs.
- Registrars’ antibiotic prescribing for acute bronchitis (72%) remains high.

## Background

Antibiotic resistance is a global health threat, with an attributable mortality of 1.27 million deaths in 2019.^1^ The number of deaths is expected to rise if efforts to reduce antibiotic prescribing are not made.^2^ The majority of antibiotics are prescribed in primary care. Inappropriate antibiotic prescribing contributes to this crisis.^3^ Inappropriate antibiotic prescribing refers to prescribing for conditions for which antibiotics provide little-to-no benefit, including self-limiting respiratory diseases such as acute bronchitis/bronchiolitis (hereafter referred to as acute bronchitis) and upper respiratory tract infections (URTIs).

In Australian general practice, antibiotics are prescribed in 81.5% of diagnoses of acute bronchitis, and in 35.8% of cases of URTI in 2019.^3^ Although overall antibiotic use in Australia is trending downwards, greater reductions are needed.^3^ Guidelines state that antibiotics are not indicated for these conditions, due to compelling evidence for lack of substantive benefit.^4,5^

General practitioner (GP) registrars (vocational trainees in general practice) are of particular interest regarding appropriate antibiotic prescribing,^6^ and comprised approximately 10% of the GP workforce in Australia by headcount in 2020.^7^ Although registrars have lower antibiotic prescribing rates than their established GP colleagues, their prescribing is still high.^5,8^ However, their antibiotic prescribing habits have been shown to be amenable to education interventions.^9,10^ The effect of these interventions could have a long-term impact as antibiotic prescribing habits established early in a clinical career may tend to persist.^11^ Previous research in an Australian GP registrar population found no reduction in individual registrars’ antibiotic prescribing for acute bronchitis or URTI as they progressed through training.^8^ However, these findings, considered together with those of an earlier cross-sectional analysis in this registrar population, suggest that there may have been some reduction over time in overall (training programme level) antibiotic prescribing for URTI.^12^

In this study, we aimed to establish trends (from 2010 to 2019) in Australian GP registrars’ antibiotic prescribing for acute bronchitis and URTI in Australia.

## Methods

### The ReCEnT project

This was a longitudinal analysis of data from the Registrars Clinical Encounters in Training (ReCEnT) from 2010 to 2019, recording content of 413,306 consultations. ReCEnT is a multicentre, ongoing cohort study of the in-consultation clinical experiences of Australian GP registrars.^13^ Before 2016, 5 of Australia’s 17 Regional Training Providers (RTPs) participated in ReCEnT. In 2016, there was a major reorganization of Australia’s GP vocational training programme, with 3 of Australia’s 9 Regional Training Organisation (RTOs) (43% of all Australian registrars^14^) subsequently participating in ReCEnT. In ReCEnT, registrars record 60 consecutive consultations (during 2010–2019, on paper case report forms) 3 times during training (once in each of their 3 6-month general practice terms). Only in-practice consultations are recorded (not home visits or nursing home visits) and not “single issue” clinics (such as influenza immunization clinics). Registrars also complete a cross-sectional questionnaire at each 6-month period. The questionnaire elicits characteristics of the GP registrar and their current practice. The methods of the ReCEnT study have been described elsewhere.^15^

### Outcome factor

The outcome factor was whether a systemic antibiotic was prescribed. Antibiotics were defined as J01 (systemic antibiotics) according to the Anatomical Therapeutic Chemical (ATC) classification system.^16^ In ReCEnT, prescription (ATC) is directly linked to the problem/diagnosis (ICPC-2 code, see below) for which the prescription is made.

### Study factor

The independent variable of interest was year (2010–2019), modelled as a continuous variable.

### Covariates

These included patient-, registrar-, practice-, and consultation-level variables:

*Registrar variables*: age, gender, full time/part time status, Australian or non-Australian medical degree, training stage (Term 1, 2, or 3).
*Patient*: age, gender, Aboriginal and/or Torres Strait Island status, non-English speaking background, new to practice, new to registrar.
*Practice*: rurality, socio-economic index for areas (SEIFA), bulk-billing (consultations are government funded with no out-of-pocket cost to patient) or non-bulk-billing, size of practice, region.
*Consultation*: pathology/imaging ordered, duration, number of issues addressed, whether or not the registrar sought information advice (i.e. supervisor/books/specialist/e-resources), referral ordered, follow-up ordered, learning goals generated).

### Statistical analyses

Two separate analyses were performed, limited to diagnoses classified as new cases of acute bronchitis (R78, in the International Classification of Primary Care [ICPC] system) and URTI (R74), respectively.^17^ We excluded subsection R74-6 from the URTI classification due to its ambiguity of “sore throat” and its potential to include misclassified conditions such as pharyngitis or tonsilitis. All analyses were at the level of problem/diagnosis (rather than consultation). The proportion of problems managed that were URTIs and acute bronchitis were each calculated with 95% confidence intervals (CIs). Proportions of acute bronchitis problems/diagnoses and of URTI problems/diagnoses that were treated with antibiotics were also calculated with 95% CIs. Descriptive analysis of the type of antibiotic prescribed for acute bronchitis and for URTI was also performed.

Descriptive statistics included frequencies for categorical variables and mean with SD for continuous variables.

A mixed-effects logistic regression model was used for the longitudinal analyses, to account for repeated measures on registrars over time. The model included a random intercept for registrar to account for nonindependence of prescribing practice within registrars. A fixed effect for time was included, representing the calendar year of the consultation, assuming linearity in the log-odds of the response. The exponentiated parameter estimate represents the estimated multiplicative change per year in the odds of prescribing an antibiotic, as a combination of between- and within-registrar effects.

All other measured variables were modelled as fixed effects. Covariates were selected for inclusion in the multivariable model by screening each covariate for association with the outcome, using a univariate model, adjusted for year of consultation. Covariates with a univariate *P* value less than 0.20 were considered for inclusion in the multiple regression model. Once the model with all significant covariates was fitted, model reduction was assessed. Covariates that were no longer significant (at *P* < 0.2) in the multivariable model were tested for removal from the model. If the removal of the covariate did not substantively change the resulting model, the covariate was removed from the final model. A substantive change to the model was defined as any covariate in the model having a change in the effect size (odds ratio, OR) of greater than 10%. The regressions modelled the log-odds that an antibiotic was prescribed for a new diagnosis of acute bronchitis/bronchiolitis. Goodness of fit was assessed by checking linearity of the effect of time, checking residuals, performing the Hosmer–Lemeshow (H–L) test, and checking for the presence of influential observations.

Analyses were programmed using STATA 16.0 and SAS V9.4. The study was approved by the Human Research Ethics Committee of the University of Newcastle: approval number H-2009-0323.

## Results

A total of 2,839 registrars recorded 413,306 consultations with a response rate of 96.1%. See Table 1 for the characteristics of participating registrars and practices; registrars were mostly trained in Australia (80%), female (63%), and the average age was 32.6 (±6.3) years.

**Table 1.** Demographic characteristics of participating registrars and practices in the ReCEnT study, 2010–2019.

	Class	Value
Registrar variable (*n*= 2,839)		
Registrar gender	Male, *n* (%)	1,077 (37.9%)
	Female, *n* (%)	1,762 (62.1%)
Qualified as doctor, overseas	Yes, *n* (%)	565 (20.0%)
	No, *n* (%)	2,261 (80.0%)
Years worked at practice previously	Mean, years (±SD)	3.4 (3.4)
Pathway registrar enrolled in	General, *n* (%)	1,960 (69.7%)
	Rural, *n* (%)	853 (30.3%)
Registrar round/practice variables (*n* = 6,954)		
Registrar age	Mean, years (±SD)	32.6 (6.3)
Registrar works, full time	Yes, *n* (%)	5,134 (76.9%)
	No, *n* (%)	1,543 (23.1%)
Registrar training term	Term 1, *n* (%)	2,691 (38.7%)
	Term 2, *n* (%)	2,477 (35.6%)
	Term 3, *n* (%)	1,786 (25.7%)
Practice rurality	Major city, *n* (%)	4,328 (62.3%)
	Inner regional, *n* (%)	1,835 (26.4%)
	Outer regional, *n* (%)	706 (10.2%)
	Remote, *n* (%)	67 (1.0%)
	Very remote, *n* (%)	16 (0.2%)
Practice SEIFA index	Mean, years (±SD)	5.4 (2.8)
Practice routinely bulk bills	Yes, *n* (%)	4,920 (71.4%)
	No, *n* (%)	1,970 (28.6%)
Larger practice (>5 GPs)	Yes, *n* (%)	4,092 (61.3%)

From 2010 to 2019, there were 28,372 new diagnoses of URTI and 5,289 new diagnoses of acute bronchitis/bronchiolitis. Antibiotics were prescribed in 13.5% (95% CI: 13.2%–14.0%) of URTI cases, and 74.6% (95% CI: 73.4%–75.8%) of acute bronchitis cases. The proportions of individual antibiotics prescribed for URTI and acute bronchitis are presented in Table 2. Amoxicillin was the most frequently prescribed antibiotic for both URTI (56% [95% CI: 54.9%, 57.7%]) and acute bronchitis (49% [95% CI: 48.0%, 50.7%]). The second most common antibiotics prescribed were phenoxymethylpenicillin for URTI (11% [95% CI: 10.1%, 11.8%]) and doxycycline for acute bronchitis (16% [95% CI: 14.6%, 16.6%]). For both conditions, amoxicillin with clavulanic acid was the third most prescribed antibiotic: URTI (9% [95% CI: 8.1%, 9.7%]) and acute bronchitis (13% [95% CI: 12.5%, 14.3%]).

**Table 2.** Proportions of antibiotics prescribed during the ReCEnT study, 2010–2019.

URTI			Acute bronchitis		
Antibiotic	Proportion (95% CI)		Antibiotic	Proportion (95% CI)	
Amoxicillin	56%	(54.9%, 57.7%)	Amoxicillin	49%	(48.0%,50.7%)
Phenoxymethylpenicillin	11%	(10.1%, 11.8%)	Doxycycline	16%	(14.6%, 16.6%)
Amoxicillin/clavulanic acid	9%	(8.1%, 9.7%)	Amoxicillin/clavulanic acid	13%	(12.5%, 14.3%)
Cefalexin	7%	(6.5%, 7.9%)	Roxithromycin	11%	(9.7%, 11.4%)
Roxithromycin	7%	(6.1%, 7.5%)	Cefalexin	4%	(3.3%, 4.4%)
Doxycycline	4%	(3.8%, 5.0%)	Clarithromycin	3%	(2.8%, 3.8%)
Other	5%	(4.8%, 6.1%)	Other	4%	(3.4%, 4.5%)

**Fig. 1.**
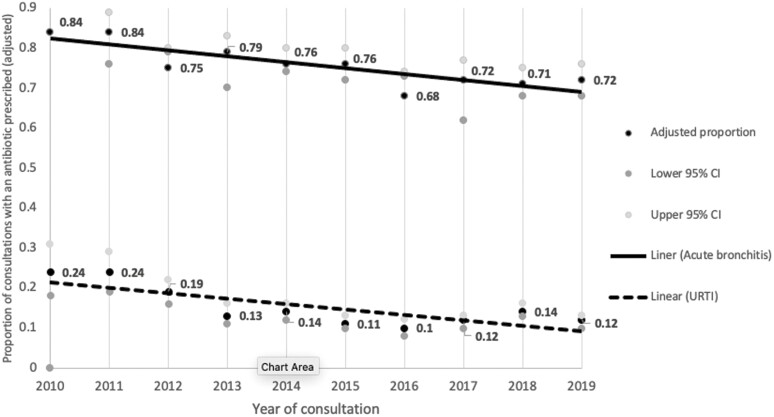
Australian general practitioner registrar proportions of consultations with an antibiotic prescribed for new diagnoses of URTIs and acute bronchitis 2010 and 2019 (adjusted).

Prescribing for both conditions decreased in the 10-year period, from 24% (95% CI: 18%, 31%) to 12% (95% CI: 10%, 13%) for URTI and 84% (95% CI: 76%, 92%) to 72% (95% CI: 68%, 76%) for acute bronchitis. The characteristics associated with prescribing of antibiotics for acute bronchitis and URTI are presented in Appendix Tables 1 and 2. On multivariable analysis, our measure of time, “Year,” was significantly associated with a decrease in prescribing in the 10-year period for both URTI (OR 0.90 [0.88, 0.93], *P* ≤ 0.001) and acute bronchitis (OR 0.92 [0.88, 0.96], *P* ≤ 0.001). See Table 3 for the multivariable regression analyses with outcome “antibiotic prescribed,” and Fig. 1 for the adjusted proportion of antibiotics prescribed for (i) URTI and (ii) acute bronchitis by year from 2010 to 2019. See Appendix Tables 3 and 4 for the univariable regression models with outcome “antibiotic prescribed.”

**Table 3.** Associations with prescribing antibiotics for URTI and acute bronchitis, adjusted models, 2010–2019 ReCEnT data.

Variable	Class	URTI		Acute bronchitis	
		OR (95% CI)	*P*	OR (95% CI)	*P*
Longitudinal factor	Year of consultation	0.90 (0.88, 0.93)	<0.001	0.92 (0.88, 0.96)	<0.001
Patient factors					
Aboriginal and/or Torres Strait islander	Yes			1.37 (0.75, 2.50)	0.31
Patient age group	5–14 years	1.48 (1.26, 1.74)	<0.001	6.54 (4.54, 9.44)	<0.001
Ref: 0–5	15–24 years	2.06 (1.76, 2.41)	<0.001	7.68 (5.32, 11.1)	<0.001
	25–44 years	2.39 (2.09, 2.73)	<0.001	6.73 (5.22, 8.69)	<0.001
	45–64 years	3.04 (2.64, 3.52)	<0.001	7.15 (5.54, 9.23)	<0.001
	65 years+	4.70 (3.93, 5.62)	<0.001	8.50 (6.46, 11.2)	<0.001
Patient/practice status	New to registrar			1.21 (1.00, 1.47)	0.048
	New to practice			1.27 (0.92, 1.76)	0.15
Registrar factors					
Qualified as doctor in Australia	Yes	0.76 (0.63, 0.92)	0.004		
Registrar FTE^a^	Part-time	0.92 (0.81, 1.05)	0.20		
Registrar age		0.99 (0.98, 1.00)	0.039	1.01 (1.00, 1.03)	0.15
Registrar gender	Female	0.91 (0.81, 1.04)	0.17		
Training term/post					
Ref: Term 1	Term 2	1.11 (1.00, 1.23)	0.046	1.35 (1.10, 1.65)	0.004
	Term 3	1.17 (1.04, 1.32)	0.008	1.24 (0.98, 1.55)	0.068
Practice factors					
Practice routinely bulk bills	Yes	0.86 (0.76, 0.98)	0.023		
Practice size	Small	1.11 (0.99, 1.23)	0.063	0.88 (0.73, 1.07)	0.21
Region	Region 2	0.88 (0.65, 1.18)	0.39	0.73 (0.47, 1.12)	0.15
Ref: region 1	Region 3	0.69 (0.52, 0.91)	0.008	0.72 (0.49, 1.05)	0.092
	Region 4	0.81 (0.68, 0.98)	0.028	1.48 (1.13, 1.92)	0.004
	Region 5	0.72 (0.42, 1.23)	0.22	1.22 (0.57, 2.60)	0.61
	Region 6	1.32 (1.04, 1.68)	0.022	1.47 (1.03, 2.11)	0.034
	Region 7	0.99 (0.75, 1.31)	0.94	0.91 (0.59, 1.39)	0.66
Rurality	Inner regional	1.12 (0.94, 1.33)	0.20	1.39 (1.06, 1.81)	0.017
	Outer regional remote	1.49 (1.15, 1.93)	0.003	1.52 (1.04, 2.24)	0.032
Consultation factors					
Consultation duration		1.02 (1.02, 1.03)	<0.001	0.98 (0.97, 1.00)	0.008
Number of problems		0.77 (0.72, 0.83)	<0.001		
Assistance sought	Other sources	5.60 (4.68, 6.69)	<0.001	2.48 (1.87, 3.30)	<0.001
	Supervisor	2.36 (1.73, 3.22)	<0.001	1.57 (1.04, 2.37)	0.033
Follow-up ordered	GP appointment with registrar	1.51 (1.36, 1.69)	<0.001	1.50 (1.24, 1.81)	<0.001
	With someone else	1.33 (1.00, 1.76)	0.051	1.40 (0.93, 2.09)	0.11
Imaging ordered	Yes	1.44 (0.92, 2.26)	0.11	1.32 (0.96, 1.80)	0.088
Learning goals generated	Yes	1.40 (1.13, 1.73)	0.002	0.89 (0.66, 1.19)	0.43
Pathology ordered	Yes	1.42 (1.19, 1.69)	<0.001		
Referral ordered	Yes			0.16 (0.09, 0.28)	<0.001

Full-time employment.

Regression diagnostics found reasonable linearity of the time effect. The model had a good fit for both URTI (H–L *χ*^2^ = 20.5, *P* = 0.30) and acute bronchitis (*χ*^2^ = 16.8, *P* = 0.54) and there were no influential observations.

## Discussion

### Principal findings

Our longitudinal analysis shows that GP registrar antibiotic prescribing for both URTI and acute bronchitis has decreased by an absolute margin of 12% in the 10-year period from 2010 to 2019; from 24% to 12% for URTI (a 50% relative reduction) and 84% to 72% for acute bronchitis. The time trend was statistically significant following adjustment for potential confounding. The most prescribed antibiotic for both conditions was amoxicillin.

### Comparisons with previous literature

Our findings are consistent with those from the Antimicrobial Use and Resistance in Australia (AURA) report, which collects deidentified data extracted from GP clinical records.^3^ The AURA report found that overall volume of systemic antibiotics fell by 3.5% in the past 5 years, with a 11.2% reduction in proportion of patients with URTI prescribed antibiotics and a 9% reduction for patients with acute bronchitis. Comparison of our findings with those of AURA suggests registrars consistently prescribe less antibiotics than the wider population of established GPs; the GP registrars had a 23% lower rate for URTI and a 10% lower antibiotic prescribing rate for acute bronchitis.^3,5^

A previous analysis of registrar antibiotic prescribing for URTI and acute bronchitis (covering the period 2010–2015) found no significant change in individual registrars’ antibiotic prescribing rate during training (that is, from Term 1 to Term 2 to Term 3).^8^ This suggests that, over the course of an individual registrar’s training, prescribing rates for these conditions are stable. Our current analysis demonstrates that there is “within-programme” temporal change (as opposed to “within-registrar” change) in antibiotic prescribing. This is consistent with other findings from the ReCEnT study examining benzodiazepine prescribing habits, i.e. within-registrar benzodiazepine prescribing patterns do not change through training, but within-programme benzodiazepine prescribing reduces significantly.^18^

### Strengths and limitations

The ReCEnT study is a unique database that describes the in-consultation clinical and educational experiences and behaviours of GPs in the earliest stages of their general practice careers. It allows in-depth analysis of prescribing habits, specifically linked to the problem/diagnosis for which prescriptions are made. Other data sources utilize national prescribing databases, which although having very high volume of information/participants, do not have the clear linkage of prescription and indication present in ReCEnT. This is a limitation of the AURA report data where GPs can voluntarily fill in “reason for prescription” which is only documented in 36.4% of consultations.^3^ The high response rate of the ReCEnT study is a major strength.^19^ Like clinical notes, ReCEnT data are requested to be recorded at the time of the consultation, therefore recall bias is minimized. Our analyses accounted for a large number of potential confounding variables such as patient age or socioeconomic status. However, a limitation was that we do not have data on patient comorbidities (noting that marked immunosuppression—affecting a very small proportion of patients—may be an indication for antibiotic prescription for acute bronchitis or URTI).

Utilizing the ICPC-2 system to link diagnoses to prescribing has limitations, as it does not indicate if the patient is experiencing more complex symptoms. Symptom severity, however, is not an indication for the prescription of antibiotics if the diagnosis is URTI or acute bronchitis. Another potential limitation is that data collection was noncontinuous (it was obtained every 6 months); this may not represent the patterns of prescribing for respiratory tract infections over an entire year (noting the seasonal variability in respiratory infections). The timing of data collection, however, was at approximately the same times each year and is unlikely to have biased our estimates of the annual temporal trend.

Differentiation of delayed and immediate prescribing is not included in this particular analysis due to lack of data on this variable in earlier data collection periods, but it has been included in ReCEnT data in later collection periods. Delayed prescribing accounts for approximately 9% of URTI prescribing, and 11% of acute bronchitis in registrars’ practice.^20^ We do not know the temporal trend in delayed versus immediate prescribing, but any change would plausibly be an increase in delayed prescribing. Any consequent potential increase in overall prescribing over time would make our findings of reduced prescribing more robust.

### Implications for practice

GP registrars prescribe considerably fewer antibiotics for both URTI and acute bronchitis compared with established GPs and prescribing rates have declined in the past 10 years. URTI antibiotic prescribing is relatively low, more in-line with current clinical guidelines. An intervention to reduce antibiotic prescribing which found no significant change in prescribing for URTI in this registrar population may, thus, have been subject to a “ceiling” effect.^10^ Registrar prescribing for acute bronchitis, however, remains inappropriately high at 72%. This represents a continuing high burden of unnecessary antibiotic use. Other countries have achieved much lower prescribing rates, and have low rates of antibiotic resistance.^21,22^ National antibiotic prescribing levels need to reduce further in order to have the desired impact on antimicrobial resistance, and this may be achieved through targeting the next generation of GPs.^23^ Previous evidence suggests that focussed education of registrars can reduce antibiotic prescribing for acute bronchitis.^10^ Wider implementation of similar educational strategies is essential.

## Conclusion

We have found significant reductions in registrars’ prescribing of antibiotics for URTI and acute bronchitis over the period 2010–2019. Prescribing for acute bronchitis, though, remains high and should be the subject of further educational interventions and antimicrobial stewardship interventions.

## Supplementary Material

cmac052_suppl_Supplementary_Appendix_Table_1

cmac052_suppl_Supplementary_Appendix_Table_2

cmac052_suppl_Supplementary_Appendix_Table_3

cmac052_suppl_Supplementary_Appendix_Table_4

cmac052_suppl_Supplementary_Checklist

## Data Availability

The data underlying this article cannot be shared publicly due to advice of the relevant Human Research Ethics Committee.
