# Supplementary material for: Antibiotic prescribing for upper respiratory tract infections and acute bronchitis: a longitudinal analysis of general practitioner trainees
Source: Fam Pract. 2022 May 28;39(6):1063–9. doi: 10.1093/fampra/cmac052 (PMC9680663; doi:10.1093/fampra/cmac052)
Supplement: cmac052_suppl_Supplementary_Appendix_Table_1 [file cmac052_suppl_supplementary_appendix_table_1.pdf]

Appendix Table 1: Characteristics associated with prescribing antibiotics for URTI over time

Variable	Class	Antibiotics prescribed (n, unadjusted proportion %)				p-value
		No		Yes		
**Longitudinal factors**						
Year of consultation	mean (SD)	8(2)		6(3)		<0.001
	2010	249	72%	97	28%	
	2011	717	77%	220	23%	
	2012	1446	81%	337	19%	
	2013	2063	88%	283	12%	
	2014	2351	88%	323	12%	
	2015	2597	89%	324	11%	
	2016	1773	90%	197	10%	
	2017	3283	88%	451	12%	
	2018	4874	85%	851	15%	
	2019	5179	87%	757	13%	
**Patient factors**						
Patient age group	0-<5 years	6065	91%	566	9%	<0.001
	05-14 years	3628	89%	433	11%	
	15-24 years	3324	87%	506	13%	
	25-44 years	6371	86%	1063	14%	
	45-64 years	3651	82%	793	18%	
	65 years+	1219	74%	426	26%	
Patient gender	Female	13668	86%	2177	14%	0.28
	Male	10383	87%	1586	13%	
Aboriginal and/or Torres Strait Islander	No	22588	87%	3521	13%	0.32
	Yes	345	84%	65	16%	
Non-english speaking background	No	20801	86%	3260	14%	0.70
	Yes	2282	87%	348	13%	
Patient/practice status	Existing patient	5399	86%	869	14%	0.62
	New to practice	2373	86%	382	14%	
	New to registrar	16345	87%	2510	13%	
**Registrar factors**						
Registrar age	Mean, years (SD)	32 (6)		33 (6)		<0.001
Registrar gender	Female	14183	87%	2138	13%	0.096
	Male	10349	86%	1702	14%	
Registrar FT or PT	Full-time	18405	86%	2962	14%	0.11
	Part-time	5345	88%	728	12%	
Term	Term 1	9338	86%	1527	14%	0.17
	Term 2	8899	87%	1385	13%	
	Term 3	6295	87%	928	13%	
Worked at practice previously	No	19664	87%	3031	13%	0.57
	Yes	4644	86%	753	14%	
Qualified as doctor in Australia	No	3695	82%	837	18%	<0.001
	Yes	20742	87%	2982	13%	
**Practice factors**						
SEIFA index	mean (SD)	6 (3)		5 (3)		0.006
Practice size	Large	14867	87%	2165	13%	0.002
	Small	8831	85%	1539	15%	
Practice routinely bulk bills	No	15966	86%	2552	14%	0.19
	Yes	8295	87%	1252	13%	
Rurality	Inner regional	5011	84%	946	16%	<0.001
	Major city	17860	88%	2533	12%	
	Outer regional remote	1657	82%	361	18%	
Training Region	Region 1	3946	83%	811	17%	<0.001

Variable	Class	Antibiotics prescribed (n, unadjusted proportion %) No		Yes		p-value
	Region 2	1360	86%	219	14%	
	Region 3	1939	87%	278	13%	
	Region 4	9455	88%	1266	12%	
	Region 5	256	82%	56	18%	
	Region 6	5577	86%	879	14%	
	Region 7	1999	86%	331	14%	
**Consultation factors**						
Consultation duration	Mean, minutes (SD)	15 (7)		15 (7)		<0.001
Number of problems	mean (SD)	1 (1)		1 (1)		0.004
Sought help any source	None	14183	87%	2138	13%	<0.001
	Other sources	10349	86%	1702	14%	
	Supervisor	18405	86%	2962	14%	
Pathology ordered	No	5345	88%	728	12%	<0.001
	Yes	9338	86%	1527	14%	
Imaging ordered	No	8899	87%	1385	13%	<0.001
	Yes	6295	87%	928	13%	
Referral ordered	No	19664	87%	3031	13%	0.075
	Yes	4644	86%	753	14%	
Follow-up ordered	GP appointment or phone	3695	82%	837	18%	<0.001
	None	20742	87%	2982	13%	
	With someone else	14183	87%	2138	13%	
Learning goals generated	No	10349	86%	1702	14%	<0.001
	Yes	18405	86%	2962	14%	
