# Supplementary material for: Antibiotic prescribing for upper respiratory tract infections and acute bronchitis: a longitudinal analysis of general practitioner trainees
Source: Fam Pract. 2022 May 28;39(6):1063–9. doi: 10.1093/fampra/cmac052 (PMC9680663; doi:10.1093/fampra/cmac052)
Supplement: cmac052_suppl_Supplementary_Appendix_Table_2 [file cmac052_suppl_supplementary_appendix_table_2.pdf]

## Appendix Table 2: Characteristics associated with prescribing antibiotics for acute bronchitis over time

| Variable | Class | Antibiotics prescribed (n, unadjusted proportion %) No | | Yes | | p-value |
|---|---|---|---|---|---|---|
| **Longitudinal factors** | | | | | | |
| Year of consultation | Mean (SD) | | | | | |
| | 2010 | 17 | 18% | 76 | 82% | <0.001 |
| | 2011 | 39 | 16% | 204 | 84% | |
| | 2012 | 85 | 23% | 285 | 77% | |
| | 2013 | 99 | 24% | 321 | 76% | |
| | 2014 | 125 | 24% | 404 | 76% | |
| | 2015 | 131 | 21% | 489 | 79% | |
| | 2016 | 105 | 31% | 233 | 69% | |
| | 2017 | 179 | 28% | 462 | 72% | |
| | 2018 | 268 | 28% | 693 | 72% | |
| | 2019 | 292 | 27% | 782 | 73% | |
| **Patient factors** | | | | | | |
| Patient age group | 0-<5 years | 551 | 59% | 388 | 41% | <0.001 |
| | 05-14 years | 78 | 21% | 295 | 79% | |
| | 15-24 years | 73 | 18% | 328 | 82% | |
| | 25-44 years | 225 | 19% | 941 | 81% | |
| | 45-64 years | 234 | 18% | 1062 | 82% | |
| | 65 years+ | 167 | 16% | 883 | 84% | |
| Patient gender | Female | 696 | 24% | 2176 | 76% | 0.098 |
| | Male | 611 | 26% | 1698 | 74% | |
| Aboriginal and/or Torres Strait Islander | No | 1218 | 25% | 3601 | 75% | 0.32 |
| | Yes | 32 | 29% | 79 | 71% | |
| Non-english speaking background | No | 1165 | 25% | 3456 | 75% | 0.75 |
| | Yes | 88 | 27% | 241 | 73% | |
| Patient/practice status | Existing patient | 400 | 28% | 1023 | 72% | 0.04 |
| | New to practice | 117 | 25% | 352 | 75% | |
| | New to registrar | 797 | 24% | 2482 | 76% | |
| **Registrar factors** | | | | | | |
| Registrar age | Mean, years (SD) | 32 | (6) | 33 | (6) | 0.017 |
| Registrar gender | Female | 784 | 25% | 2390 | 75% | 0.43 |
| | Male | 556 | 26% | 1559 | 74% | |
| Registrar FT or PT | Full-time | 1020 | 26% | 2967 | 74% | 0.47 |
| | Part-time | 272 | 25% | 801 | 75% | |
| Term | Term 1 | 532 | 28% | 1387 | 72% | 0.003 |
| | Term 2 | 469 | 23% | 1537 | 77% | |
| | Term 3 | 339 | 25% | 1025 | 75% | |
| Worked at practice previously | No | 1055 | 26% | 2999 | 74% | 0.056 |
| | Yes | 268 | 23% | 898 | 77% | |
| Qualified as doctor in Australia | No | 274 | 23% | 905 | 77% | 0.071 |
| | Yes | 1062 | 26% | 3023 | 74% | |
| **Practice factors** | | | | | | |
| SEIFA index | Mean (SD) | 6 | (3) | 6 | (3) | 0.84 |
| Practice size | Large | 800 | 24% | 2489 | 76% | 0.065 |
| | Small | 493 | 27% | 1307 | 73% | |
| Practice routinely bulk bills | No | 980 | 25% | 2921 | 75% | 0.81 |
| | Yes | 353 | 26% | 998 | 74% | |
| Rurality | Inner regional | 336 | 23% | 1141 | 77% | 0.042 |
| | Major city | 873 | 27% | 2399 | 73% | |

| Variable | Class | Antibiotics prescribed (n, unadjusted proportion %) | | | | p-value |
|---|---|---|---|---|---|---|
| | | No | | Yes | | |
| Training Region | Outer regional remote | 131 | 24% | 409 | 76% | 0.20 |
| | Region 1 | 83 | 28% | 209 | 72% | |
| | Region 2 | 143 | 27% | 383 | 73% | |
| | Region 3 | 411 | 22% | 1461 | 78% | |
| | Region 4 | 24 | 22% | 87 | 78% | |
| | Region 5 | 241 | 29% | 586 | 71% | |
| | Region 6 | 126 | 28% | 328 | 72% | |
| | Region 7 | 312 | 26% | 895 | 74% | |
| **Consultation factors** | | | | | | |
| Consultation duration | Mean, minutes (SD) | 18 | (8) | (17) | (7) | <0.001 |
| Number of problems | mean (SD) | 1 | (1) | (1 | (1) | 0.99 |
| Sought help any source | None | 1095 | 26% | 3108 | 74% | <0.001 |
| | Other sources | 149 | 18% | 665 | 82% | |
| | Supervisor | 96 | 35% | 176 | 65% | |
| Pathology ordered | No | 1220 | 26% | 3500 | 74% | 0.009 |
| | Yes | 120 | 21% | 449 | 79% | |
| Imaging ordered | No | 1250 | 27% | 3453 | 73% | <0.001 |
| | Yes | 90 | 15% | 496 | 85% | |
| Referral ordered | No | 1243 | 24% | 3918 | 76% | <0.001 |
| | Yes | 97 | 76% | 31 | 24% | |
| Follow-up ordered | GP appointment or phone | 548 | 23% | 1836 | 77% | 0.002 |
| | None | 723 | 28% | 1903 | 72% | |
| | With someone else | 69 | 25% | 210 | 75% | |
| Learning goals generated | No | 1077 | 25% | 3258 | 75% | 0.087 |
| | Yes | 175 | 28% | 446 | 72% | |
