# Supplementary material for: Antibiotic prescribing for upper respiratory tract infections and acute bronchitis: a longitudinal analysis of general practitioner trainees
Source: Fam Pract. 2022 May 28;39(6):1063–9. doi: 10.1093/fampra/cmac052 (PMC9680663; doi:10.1093/fampra/cmac052)
Supplement: cmac052_suppl_Supplementary_Appendix_Table_3 [file cmac052_suppl_supplementary_appendix_table_3.pdf]

Appendix Table 3: Univariate and multivariable analysis for upper respiratory tract infections antibiotic prescribing

URTI		Univariate model (all covariates model) Adjusted model		Multivariable model (with significant univariates) Adjusted model	
**Variable**	**Class**	**OR (95% CI)**	**p-value**	**OR (95% CI)**	**p-value**
**Longitudinal factor**					
Year of consultation		0.91 (0.88, 0.93)	<0.001	0.90 (0.88, 0.93)	<0.001
**Patient factors**					
Aboriginal and/or Torres Strait Islander	Yes	0.97 (0.66, 1.41)	0.86		
Non-english speaking background	Yes	1.03 (0.87, 1.21)	0.74		
Patient gender	Female	0.97 (0.89, 1.07)	0.58		
Patient age group	05-14 years	1.48 (1.25, 1.75)	<0.001	1.49 (1.27, 1.74)	<0.001
	15-24 years	2.06 (1.75, 2.43)	<0.001	2.06 (1.76, 2.41)	<0.001
	25-44 years	2.36 (2.05, 2.72)	<0.001	2.39 (2.09, 2.73)	<0.001
	45-64 years	3.03 (2.61, 3.53)	<0.001	3.05 (2.64, 3.52)	<0.001
	65 years+	4.70 (3.90, 5.68)	<0.001	4.70 (3.93, 5.63)	<0.001
Patient/practice status	New to registrar	1.00 (0.89, 1.11)	0.95		
	New to practice	1.06 (0.89, 1.26)	0.53		
**Registrar factors**					
Qualified as doctor in Australia	Yes	0.72 (0.59, 0.87)	0.001	0.76 (0.63, 0.92)	0.004
Registrar FTE*	Part-time	0.88 (0.77, 1.01)	0.072	0.92 (0.81, 1.04)	0.19
Registrar age		0.99 (0.97, 1.00)	0.022	0.99 (0.98, 1.00)	0.037
Registrar gender	Female	0.91 (0.80, 1.04)	0.16	0.91 (0.81, 1.04)	0.17
Training term/post	Term 2	1.17 (1.03, 1.32)	0.013	1.11 (1.00, 1.23)	0.05
	Term 3	1.23 (1.08, 1.40)	0.002	1.17 (1.04, 1.32)	0.009
Worked at practice previously	Yes	0.94 (0.81, 1.08)	0.37		
**Practice factors**					
Practice routinely bulk bills	Yes	0.87 (0.76, 1.00)	0.048	0.87 (0.76, 0.99)	0.035
Practice size	Small	1.10 (0.99, 1.24)	0.079	1.11 (0.99, 1.23)	0.064
Training Region	Region 2	0.92 (0.68, 1.25)	0.6	0.88 (0.65, 1.18)	0.38
	Region 3	0.72 (0.54, 0.96)	0.028	0.69 (0.52, 0.91)	0.008
	Region 4	0.80 (0.66, 0.97)	0.026	0.81 (0.67, 0.97)	0.024
	Region 5	0.73 (0.42, 1.28)	0.27	0.71 (0.41, 1.21)	0.21
	Region 6	1.28 (1.00, 1.65)	0.054	1.32 (1.03, 1.68)	0.025
	Region 7	0.97 (0.72, 1.30)	0.82	0.99 (0.75, 1.31)	0.93
Rurality	Inner regional	1.12 (0.93, 1.34)	0.23	1.13 (0.95, 1.34)	0.18
	Outer regional remote	1.43 (1.08, 1.89)	0.012	1.51 (1.16, 1.98)	0.003
SEIFA Index		1.00 (0.98, 1.02)	0.98	1.01 (0.98, 1.03)	0.62
**Consultation factors**					
Consultation duration		1.02 (1.02, 1.03)	<0.001	1.02 (1.02, 1.03)	<0.001
Number of problems		0.77 (0.71, 0.82)	<0.001	0.77 (0.72, 0.83)	<0.001
Sought assistance	Other sources	5.87 (4.86, 7.08)	<0.001	5.60 (4.68, 6.69)	<0.001
	Supervisor	2.41 (1.72, 3.36)	<0.001	2.35 (1.72, 3.21)	<0.001
**Consultation outcome factors**					
Follow up ordered	GP appointment or phone	1.51 (1.35, 1.69)	<0.001	1.52 (1.36, 1.69)	<0.001
	With someone else	1.35 (1.00, 1.81)	0.047	1.33 (1.00, 1.77)	0.05
Imaging ordered	Yes	1.44 (0.90, 2.31)	0.13	1.44 (0.92, 2.26)	0.11
Learning goals generated	Yes	1.50 (1.20, 1.88)	<0.001	1.39 (1.12, 1.73)	0.002
Pathology ordered	Yes	1.38 (1.14, 1.66)	<0.001	1.41 (1.18, 1.69)	<0.001
Referral ordered	Yes	1.07 (0.54, 2.12)	0.84	1.19 (0.62, 2.27)	0.60
*FTE= full time employment					
