# Supplementary material for: Antibiotic prescribing for upper respiratory tract infections and acute bronchitis: a longitudinal analysis of general practitioner trainees
Source: Fam Pract. 2022 May 28;39(6):1063–9. doi: 10.1093/fampra/cmac052 (PMC9680663; doi:10.1093/fampra/cmac052)
Supplement: cmac052_suppl_Supplementary_Appendix_Table_4 [file cmac052_suppl_supplementary_appendix_table_4.pdf]

Appendix Table 4: Univariate and multivariable analyses: acute bronchitis

**Acute bronchitis**		**Univariate model (all covariates model) Adjusted model**		**Multivariable model (with significant univariates) Adjusted model**	
**Variable**	**Class**	**OR (95% CI)**	**p-value**	**OR (95% CI)**	**p-value**
Longitudinal factor					
Year of consultation		0.92 (0.88, 0.96)	<0.001	0.91 (0.87, 0.95)	<0.001
Patient factors					
Aboriginal and/or Torres Strait Islander	Yes	1.35 (0.74, 2.49)	0.33	1.34 (0.73, 2.46)	0.35
Non-english speaking backgroun	Yes	1.01 (0.70, 1.45)	0.97		
Patient gender	Female	0.90 (0.75, 1.07)	0.23	0.92 (0.78, 1.10)	0.37
Patient age group	05-14 years	6.73 (4.61, 9.83)	<0.001	6.46 (4.46, 9.37)	<0.001
	15-24 years	8.01 (5.45, 11.8)	<0.001	7.75 (5.32, 11.3)	<0.001
	25-44 years	7.00 (5.36, 9.15)	<0.001	6.59 (5.08, 8.55)	<0.001
	45-64 years	7.48 (5.71, 9.80)	<0.001	7.13 (5.49, 9.26)	<0.001
	65 years+	8.71 (6.53, 11.6)	<0.001	8.31 (6.27, 11.0)	<0.001
Patient/practice status	New to registrar	1.22 (1.00, 1.49)	0.056	1.25 (1.03, 1.53)	0.023
	New to practice	1.31 (0.93, 1.85)	0.12	1.36 (0.97, 1.90)	0.074
Registrar factors					
Qualified as doctor in Australia	Yes	0.94 (0.70, 1.26)	0.68	0.92 (0.69, 1.23)	0.59
Registrar FTE*	Part-time	0.96 (0.76, 1.22)	0.75		
Registrar age		1.01 (0.99, 1.03)	0.19	1.01 (0.99, 1.03)	0.20
Registrar gender	Female	1.16 (0.95, 1.42)	0.15		
Training term/post	Term 2	1.30 (1.02, 1.64)	0.031	1.30 (1.03, 1.64)	0.026
	Term 3	1.22 (0.96, 1.55)	0.11	1.21 (0.95, 1.54)	0.12
Worked at practice previously	Yes	1.14 (0.88, 1.47)	0.34	1.13 (0.87, 1.46)	0.35
Practice factors					
Practice routinely bulk bills	Yes	1.10 (0.86, 1.42)	0.44		
Practice size	Small	0.86 (0.71, 1.05)	0.14	0.89 (0.73, 1.07)	0.22
Training Region	Region 2	0.73 (0.47, 1.14)	0.17	0.75 (0.48, 1.16)	0.20
	Region 3	0.76 (0.51, 1.12)	0.16	0.76 (0.51, 1.11)	0.15
	Region 4	1.54 (1.16, 2.05)	0.003	1.55 (1.18, 2.03)	0.002
	Region 5	1.27 (0.58, 2.75)	0.55	1.24 (0.58, 2.67)	0.58
	Region 6	1.40 (0.94, 2.07)	0.095	1.52 (1.06, 2.19)	0.024
	Region 7	0.96 (0.62, 1.48)	0.84	0.96 (0.62, 1.47)	0.84
Rurality	Inner regional	1.30 (0.98, 1.73)	0.074	1.29 (0.97, 1.70)	0.08
	Outer regional remote	1.40 (0.92, 2.13)	0.12	1.43 (0.96, 2.12)	0.08
SEIFA Index		0.99 (0.96, 1.03)	0.79		
Consultation factors					
Consultation duration		0.99 (0.97, 1.00)	0.066		
Number of problems		0.92 (0.81, 1.04)	0.18		
Sought assistance	Other sources	2.46 (1.83, 3.30)	<0.001	2.52 (1.88, 3.36)	<0.001
	Supervisor	1.57 (1.02, 2.42)	0.04	1.60 (1.04, 2.45)	0.031
Consultation outcome factors					
Follow-up ordered	GP appointment or phone	1.51 (1.25, 1.84)	<0.001	1.50 (1.24, 1.82)	<0.001
	With someone else	1.32 (0.87, 2.01)	0.19	1.36 (0.90, 2.06)	0.143
Imaging ordered	Yes	1.31 (0.94, 1.82)	0.11	1.32 (0.95, 1.82)	0.098
Learning goals generated	Yes	0.85 (0.62, 1.15)	0.29	0.89 (0.65, 1.20)	0.43
Pathology ordered	Yes	1.00 (0.74, 1.36)	0.99	1.01 (0.74, 1.36)	0.97
Referral ordered	Yes	0.15 (0.08, 0.28)	<0.001	0.15 (0.08, 0.28)	<0.001

*FTE= full time employment
